# *catena*-Poly[[lead(II)-μ-*N*′-[1-(pyridin-2-yl-κ*N*)ethyl­idene]isonicotino­hydrazidato-κ^3^ *N*′,*O*:*N* ^1^] perchlorate]

**DOI:** 10.1107/S1600536811046691

**Published:** 2011-11-09

**Authors:** Gholam Hossein Shahverdizadeh, Edward R. T. Tiekink, Babak Mirtamizdoust

**Affiliations:** a Department of Chemistry, Faculty of Science, Tabriz Branch, Islamic Azad University, PO Box 1655, Tabriz, Iran; b Department of Chemistry, University of Malaya, 50603 Kuala Lumpur, Malaysia; c Department of Inorganic Chemistry, Faculty of Chemistry, University of Tabriz, PO Box 5166616471, Tabriz, Iran

## Abstract

The Pb^II^ atom in the polymeric title compound, {[Pb(C_13_H_11_N_4_O)]ClO_4_}_*n*_, is coordinated by the *N*′-[1-(pyridin-2-yl-κ*N*)ethyl­idene]isonicotinohydrazidate ligand *via* its *O*,*N*,*N*′-donors and simultaneously bridged by a neighbouring ligand *via* the pyridin-2-yl N atom. The resultant supra­molecular chain is a zigzag along the *a* axis. The stereochemistry of the Pb^II^ atom is defined by an N_3_O*E* donor set (*E* = lone pair of electrons), which results in a Ψ-trigonal–bipyramidal coordination with the O and pyridin-2-yl N atoms in axial positions. The dihedral angle between the pyridine rings of the ligand is 6.3 (3)°. The supra­molecular cationic chains are linked into a three-dimensional array *via* secondary Pb⋯O [3.133 (6) and 3.28 (7) Å] and Pb⋯N [3.028 (4) Å] inter­actions. Weak C—H⋯O inter­actions and aromatic π–π stacking [centroid–centroid separation = 3.693 (2) Å] also occur in the crystal.

## Related literature

For the structures of metal complexes containing the *N*′-[1-(2-pyrid­yl)ethyl­idene]isonicotinohydrazide ligand, see: Maurya *et al.* (2002 ▶); Abboud *et al.* (2007 ▶); Zhang & Liu (2009 ▶); Hao *et al.* (2010 ▶). For specialized crystallization techniques, see: Harrowfield *et al.* (1996 ▶).
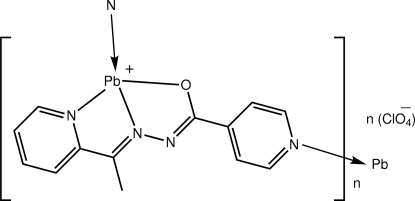


## Experimental

### 

#### Crystal data

[Pb(C_13_H_11_N_4_O)]ClO_4_
*M* *_r_* = 545.90
Monoclinic, 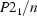

*a* = 10.0620 (6) Å
*b* = 14.4431 (8) Å
*c* = 11.1456 (7) Å
β = 99.174 (1)°
*V* = 1599.03 (16) Å^3^
*Z* = 4
Mo *K*α radiation
μ = 10.75 mm^−1^
*T* = 293 K
0.29 × 0.11 × 0.10 mm

#### Data collection

Bruker SMART CCD diffractometer
Absorption correction: multi-scan (*SADABS*; Sheldrick, 1996 ▶) *T*_min_ = 0.691, *T*_max_ = 1.000
8396 measured reflections
2811 independent reflections
2392 reflections with *I* > 2σ(*I*)
*R*_int_ = 0.026

#### Refinement

*R*[*F* ^2^ > 2σ(*F* ^2^)] = 0.022
*wR*(*F* ^2^) = 0.056
*S* = 1.07
2811 reflections
218 parameters
H-atom parameters constrained
Δρ_max_ = 0.55 e Å^−3^
Δρ_min_ = −0.45 e Å^−3^

### 

Data collection: *SMART* (Bruker, 2007 ▶); cell refinement: *SAINT* (Bruker, 2007 ▶); data reduction: *SAINT*; program(s) used to solve structure: *SHELXS97* (Sheldrick, 2008 ▶); program(s) used to refine structure: *SHELXL97* (Sheldrick, 2008 ▶); molecular graphics: *ORTEP-3* (Farrugia, 1997 ▶) and *DIAMOND* (Brandenburg, 2006 ▶); software used to prepare material for publication: *publCIF* (Westrip, 2010 ▶).

## Supplementary Material

Crystal structure: contains datablock(s) global, I. DOI: 10.1107/S1600536811046691/hb6465sup1.cif

Structure factors: contains datablock(s) I. DOI: 10.1107/S1600536811046691/hb6465Isup2.hkl

Additional supplementary materials:  crystallographic information; 3D view; checkCIF report

## Figures and Tables

**Table 1:** Selected bond lengths (Å)

Pb—O1	2.405 (3)
Pb—N1	2.597 (4)
Pb—N2	2.456 (4)
Pb—N4^i^	2.472 (4)

Symmetry code: (i) 
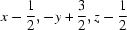
.

**Table 2:** Hydrogen-bond geometry (Å, °)

*D*—H⋯*A*	*D*—H	H⋯*A*	*D*⋯*A*	*D*—H⋯*A*
C3—H3⋯O3^ii^	0.93	2.53	3.422 (10)	160
C4—H4⋯O1^iii^	0.93	2.45	3.277 (7)	148
C10—H10⋯O2^iv^	0.93	2.57	3.485 (8)	170

Symmetry codes: (ii) 
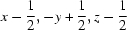
; (iii) 
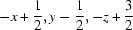
; (iv) 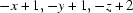.

**Fig. 1.**
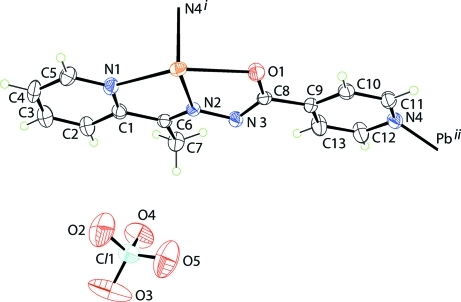
The asymmetric unit of (I) showing displacement ellipsoids at the 35% probability level. The molecular structure has been expanded to indicate the µ2-bridging mode of the tetradentate ligand. Symmetry operation i: -1/2 + x, 1.5 - y, -1/2 + z; ii: 1/2 + x, 1.5 - y, 1/2 + z.

**Fig. 2.**
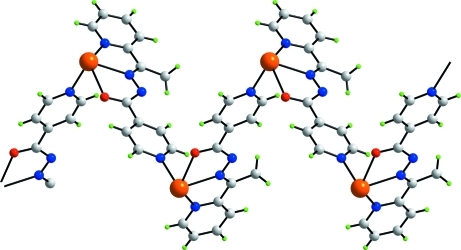
A view of the zigzag chain along the a axis in (I).

**Fig. 3.**
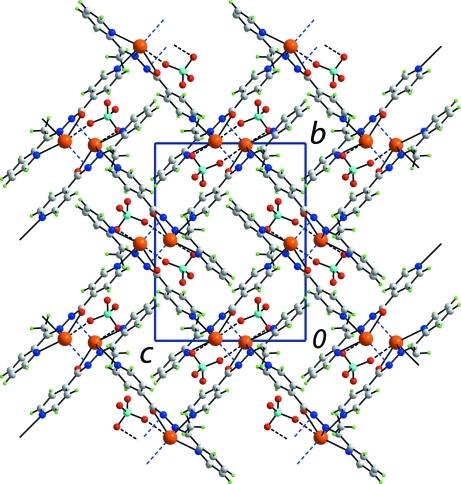
A view in projection down the a axis of the crystal packing in (I). The weaker Pb···O and Pb···N interactions (see text) are shown as black and blue dashed lines, respectively.

## supplementary crystallographic information

### Comment

Structural studies of coordination complexes containing the *N*'-[1-(2-pyridyl)ethylidene]isonicotinohydrazide ligand are rare (Maurya *et al.*, 2002; Abboud *et al.*, 2007; Zhang & Liu, 2009; Hao *et al.*, 2010). In each of these, the ligand coordinates in a tridentate mode with the terminal 4-pyridyl-N atom being non-coordinating. In the title lead(II) complex, (I), all four donor atoms participate in coordination of the Pb atom.

The asymmetric unit of (I), Fig. 1, comprises a Pb atom, a *N*'-[1-(2-pyridyl)ethylidene]isonicotinohydrazide anion and a perchlorate anion. The *N*'-[1-(2-pyridyl)ethylidene]isonicotinohydrazide ligand coordinates a lead atom in a tridentate mode, *via* the N1, N2 and O1 atoms, and simultaneously bridges a symmetry related lead atom *via* the 4-pyridyl-N4 atom, Table 1.

The resultant N_3_O donor set plus the lone pair of electrons is based on a trigonal bipyramid with the O1 and N1 atoms in axial positions [O1—Pb—N1 = 126.27 (12)°] and the remaining N atoms [N2—Pb—N4^i^ = 90.17 (13)°] and lone pair in equatorial positions; symmetry operation *i*: -1/2 + *x*, 1.5 - *y*, -1/2 + *z*. The µ_2_-bridging mode of the tetradentate *N*'-[1-(2-pyridyl)ethylidene]isonicotinohydrazide ligand leads to a zigzag chain along the *a* axis, Fig. 2. The considerable distortions from the ideal geometry arise from the acute chelate angles (O1—Pb—N2 = 64.75 (12)° and N1—Pb—N2 = 63.45 (12)°) as well as the close approach of other donor atoms. Most notable amongst the latter are Pb···O(perchlorate) interactions with the two shortest contacts being Pb···O4^ii^ of 3.133 (6) Å and Pb···O5^iii^ = 3.287 (7) Å for *i*: -1 + *x*, *y*, *z* and *ii*: 1 - *x*, 1 - *y*, 2 - *z*. These interactions along with Pb···N3^iii^ contacts of 3.028 (4) Å [*iii*: 1 - *x*, 1 - *y*, 2 - *z*] generate a three-dimensional architecture, Fig. 3.

### Experimental

A solution of methyl 2-pyridyl ketone (10 mmol) in MeOH (25 ml) was added drop wise to a solution of 4-pyridinecarboxylic acid hydrazide (10 mmol) in MeOH (15 ml). The mixture was refluxed for 6 h. The white precipitate was removed by filtration and recrystallized from MeOH solution. Then the ligand (1 mmol) was placed in one arm of a branched tube (Harrowfield *et al.*, 1996) and a mixture of lead(II) acetate (1 mmol) and sodium perchlorate (1 mmol) in the other. Methanol was then added to fill both arms, the tube sealed and the ligand-containing arm immersed in a bath at 333 K, while the other was left at ambient temperature. After 1 week, yellow needles of (I) had deposited in the arm held at ambient temperature. They were then filtered off, washed with acetone and ether, and air dried. Yield: 75%. *M*.pt.: 506 K

### Refinement

Carbon-bound H-atoms were placed in calculated positions [C—H 0.93–0.96 Å, *U*_iso_(H) = 1.2–1.5*U*_eq_(parent atom)] and were included in the refinement in the riding model approximation.

### Crystal data

**Table:**

[Pb(C_13_H_11_N_4_O)]ClO_4_	*F*(000) = 1024
*M**_r_* = 545.90	*D*_x_ = 2.268 Mg m^−^^3^
Monoclinic, *P*2_1_/*n*	Mo *K*α radiation, λ = 0.71073 Å
Hall symbol: -P 2yn	Cell parameters from 4195 reflections
*a* = 10.0620 (6) Å	θ = 2.3–25.0°
*b* = 14.4431 (8) Å	µ = 10.75 mm^−^^1^
*c* = 11.1456 (7) Å	*T* = 293 K
β = 99.174 (1)°	Needle, yellow
*V* = 1599.03 (16) Å^3^	0.29 × 0.11 × 0.10 mm
*Z* = 4	

### Data collection

**Table:**

Bruker SMART CCD diffractometer	2811 independent reflections
Radiation source: fine-focus sealed tube	2392 reflections with *I* > 2σ(*I*)
graphite	*R*_int_ = 0.026
φ and ω scans	θ_max_ = 25.0°, θ_min_ = 2.3°
Absorption correction: multi-scan (*SADABS*; Sheldrick, 1996)	*h* = −11→11
*T*_min_ = 0.691, *T*_max_ = 1.000	*k* = −11→17
8396 measured reflections	*l* = −13→13

### Refinement

**Table:**

Refinement on *F*^2^	Primary atom site location: structure-invariant direct methods
Least-squares matrix: full	Secondary atom site location: difference Fourier map
*R*[*F*^2^ > 2σ(*F*^2^)] = 0.022	Hydrogen site location: inferred from neighbouring sites
*wR*(*F*^2^) = 0.056	H-atom parameters constrained
*S* = 1.07	*w* = 1/[σ^2^(*F*_o_^2^) + (0.0241*P*)^2^ + 1.6546*P*] where *P* = (*F*_o_^2^ + 2*F*_c_^2^)/3
2811 reflections	(Δ/σ)_max_ = 0.001
218 parameters	Δρ_max_ = 0.55 e Å^−^^3^
0 restraints	Δρ_min_ = −0.45 e Å^−^^3^

### Special details

**Table:**

Geometry. All s.u.'s (except the s.u. in the dihedral angle between two l.s. planes) are estimated using the full covariance matrix. The cell s.u.'s are taken into account individually in the estimation of s.u.'s in distances, angles and torsion angles; correlations between s.u.'s in cell parameters are only used when they are defined by crystal symmetry. An approximate (isotropic) treatment of cell s.u.'s is used for estimating s.u.'s involving l.s. planes.
Refinement. Refinement of *F*^2^ against ALL reflections. The weighted *R*-factor *wR* and goodness of fit *S* are based on *F*^2^, conventional *R*-factors *R* are based on *F*, with *F* set to zero for negative *F*^2^. The threshold expression of *F*^2^ > 2σ(*F*^2^) is used only for calculating *R*-factors(gt) *etc*. and is not relevant to the choice of reflections for refinement. *R*-factors based on *F*^2^ are statistically about twice as large as those based on *F*, and *R*- factors based on ALL data will be even larger.

### Fractional atomic coordinates and isotropic or equivalent isotropic displacement parameters (Å^2^)

**Table:**

	*x*	*y*	*z*	*U*_iso_*/*U*_eq_	
Pb	0.260829 (17)	0.509355 (12)	0.899845 (16)	0.03242 (8)	
O1	0.3478 (3)	0.6440 (2)	1.0129 (3)	0.0444 (9)	
N1	0.3495 (4)	0.4394 (3)	0.7130 (4)	0.0376 (10)	
N2	0.4858 (4)	0.5592 (3)	0.8673 (3)	0.0308 (9)	
N3	0.5527 (4)	0.6241 (3)	0.9458 (4)	0.0350 (10)	
N4	0.6569 (4)	0.8756 (3)	1.2492 (4)	0.0388 (10)	
C1	0.4775 (5)	0.4584 (3)	0.7001 (4)	0.0341 (11)	
C2	0.5405 (6)	0.4140 (4)	0.6142 (5)	0.0435 (13)	
H2	0.6291	0.4280	0.6070	0.052*	
C3	0.4696 (7)	0.3487 (4)	0.5400 (5)	0.0528 (15)	
H3	0.5103	0.3183	0.4818	0.063*	
C4	0.3396 (6)	0.3285 (4)	0.5516 (5)	0.0511 (15)	
H4	0.2907	0.2843	0.5021	0.061*	
C5	0.2827 (6)	0.3754 (4)	0.6388 (5)	0.0465 (13)	
H5	0.1940	0.3621	0.6466	0.056*	
C6	0.5476 (5)	0.5291 (3)	0.7823 (4)	0.0303 (10)	
C7	0.6833 (5)	0.5647 (4)	0.7653 (5)	0.0457 (13)	
H7A	0.6929	0.6278	0.7926	0.068*	
H7B	0.6918	0.5617	0.6808	0.068*	
H7C	0.7521	0.5275	0.8117	0.068*	
C8	0.4712 (5)	0.6637 (3)	1.0126 (4)	0.0310 (11)	
C9	0.5353 (5)	0.7392 (3)	1.0933 (4)	0.0328 (11)	
C10	0.4698 (5)	0.7752 (3)	1.1837 (5)	0.0367 (11)	
H10	0.3848	0.7541	1.1932	0.044*	
C11	0.5342 (5)	0.8432 (3)	1.2592 (5)	0.0389 (12)	
H11	0.4903	0.8675	1.3196	0.047*	
C12	0.7181 (6)	0.8406 (4)	1.1623 (6)	0.0511 (15)	
H12	0.8036	0.8621	1.1550	0.061*	
C13	0.6599 (5)	0.7735 (4)	1.0819 (5)	0.0492 (14)	
H13	0.7048	0.7518	1.0207	0.059*	
Cl1	0.95237 (15)	0.36424 (10)	0.81424 (16)	0.0580 (4)	
O2	0.8284 (6)	0.3288 (5)	0.7644 (6)	0.123 (2)	
O3	1.0468 (7)	0.2953 (5)	0.8172 (8)	0.166 (4)	
O4	0.9936 (6)	0.4365 (4)	0.7458 (5)	0.1034 (19)	
O5	0.9435 (7)	0.3990 (5)	0.9303 (6)	0.136 (3)	

### Atomic displacement parameters (Å^2^)

**Table:**

	*U*^11^	*U*^22^	*U*^33^	*U*^12^	*U*^13^	*U*^23^
Pb	0.02641 (11)	0.03361 (12)	0.03622 (12)	−0.00167 (8)	0.00186 (8)	0.00472 (8)
O1	0.024 (2)	0.051 (2)	0.058 (2)	−0.0028 (16)	0.0077 (17)	−0.0170 (18)
N1	0.038 (2)	0.035 (2)	0.039 (2)	−0.0048 (18)	0.0035 (19)	−0.0047 (18)
N2	0.026 (2)	0.028 (2)	0.037 (2)	0.0003 (16)	−0.0002 (18)	−0.0026 (17)
N3	0.032 (2)	0.031 (2)	0.040 (2)	−0.0021 (17)	0.0030 (19)	−0.0055 (18)
N4	0.033 (2)	0.034 (2)	0.048 (3)	−0.0015 (18)	0.002 (2)	−0.0085 (19)
C1	0.042 (3)	0.026 (2)	0.033 (3)	0.000 (2)	0.004 (2)	0.003 (2)
C2	0.050 (3)	0.039 (3)	0.044 (3)	−0.005 (2)	0.016 (3)	−0.008 (2)
C3	0.071 (4)	0.043 (3)	0.047 (3)	−0.005 (3)	0.018 (3)	−0.011 (3)
C4	0.069 (4)	0.043 (3)	0.038 (3)	−0.017 (3)	0.001 (3)	−0.013 (3)
C5	0.038 (3)	0.044 (3)	0.055 (3)	−0.012 (2)	0.000 (3)	−0.006 (3)
C6	0.029 (3)	0.029 (2)	0.033 (3)	0.001 (2)	0.003 (2)	0.002 (2)
C7	0.041 (3)	0.052 (3)	0.046 (3)	−0.010 (3)	0.015 (3)	−0.008 (3)
C8	0.030 (3)	0.028 (2)	0.033 (3)	0.0022 (19)	−0.002 (2)	0.0010 (19)
C9	0.033 (3)	0.029 (3)	0.034 (3)	0.000 (2)	−0.001 (2)	−0.004 (2)
C10	0.030 (3)	0.037 (3)	0.043 (3)	0.000 (2)	0.005 (2)	−0.004 (2)
C11	0.037 (3)	0.040 (3)	0.040 (3)	0.001 (2)	0.008 (2)	−0.007 (2)
C12	0.036 (3)	0.051 (3)	0.070 (4)	−0.011 (3)	0.018 (3)	−0.023 (3)
C13	0.040 (3)	0.052 (3)	0.059 (4)	−0.011 (3)	0.019 (3)	−0.020 (3)
Cl1	0.0411 (8)	0.0515 (9)	0.0836 (12)	−0.0050 (7)	0.0165 (8)	0.0117 (8)
O2	0.070 (4)	0.162 (6)	0.140 (5)	−0.055 (4)	0.026 (4)	−0.042 (5)
O3	0.128 (6)	0.143 (6)	0.246 (9)	0.083 (5)	0.084 (6)	0.103 (6)
O4	0.114 (5)	0.073 (3)	0.132 (5)	−0.007 (3)	0.046 (4)	0.040 (3)
O5	0.134 (6)	0.191 (7)	0.090 (4)	−0.061 (5)	0.035 (4)	−0.037 (5)

### Geometric parameters (Å, °)

**Table:**

Pb—O1	2.405 (3)	C4—H4	0.9300
Pb—N1	2.597 (4)	C5—H5	0.9300
Pb—N2	2.456 (4)	C6—C7	1.499 (6)
Pb—N4^i^	2.472 (4)	C7—H7A	0.9600
O1—C8	1.274 (5)	C7—H7B	0.9600
N1—C1	1.347 (6)	C7—H7C	0.9600
N1—C5	1.347 (6)	C8—C9	1.494 (6)
N2—C6	1.289 (6)	C9—C13	1.372 (7)
N2—N3	1.383 (5)	C9—C10	1.389 (7)
N3—C8	1.322 (6)	C10—C11	1.385 (7)
N4—C12	1.329 (6)	C10—H10	0.9300
N4—C11	1.341 (6)	C11—H11	0.9300
N4—Pb^ii^	2.472 (4)	C12—C13	1.385 (7)
C1—C2	1.387 (7)	C12—H12	0.9300
C1—C6	1.475 (7)	C13—H13	0.9300
C2—C3	1.376 (7)	Cl1—O3	1.372 (6)
C2—H2	0.9300	Cl1—O2	1.380 (5)
C3—C4	1.367 (8)	Cl1—O4	1.394 (5)
C3—H3	0.9300	Cl1—O5	1.404 (6)
C4—C5	1.381 (7)		
			
O1—Pb—N2	64.75 (12)	N2—C6—C7	122.3 (4)
O1—Pb—N4^i^	83.77 (14)	C1—C6—C7	120.9 (4)
N2—Pb—N4^i^	90.17 (13)	C6—C7—H7A	109.5
O1—Pb—N1	126.27 (12)	C6—C7—H7B	109.5
N2—Pb—N1	63.45 (12)	H7A—C7—H7B	109.5
N4^i^—Pb—N1	83.07 (13)	C6—C7—H7C	109.5
C8—O1—Pb	116.9 (3)	H7A—C7—H7C	109.5
C1—N1—C5	117.8 (4)	H7B—C7—H7C	109.5
C1—N1—Pb	117.6 (3)	O1—C8—N3	126.7 (4)
C5—N1—Pb	123.9 (3)	O1—C8—C9	119.3 (4)
C6—N2—N3	116.7 (4)	N3—C8—C9	114.1 (4)
C6—N2—Pb	125.0 (3)	C13—C9—C10	118.6 (4)
N3—N2—Pb	118.2 (3)	C13—C9—C8	121.4 (4)
C8—N3—N2	111.5 (4)	C10—C9—C8	120.0 (4)
C12—N4—C11	117.9 (4)	C11—C10—C9	118.4 (5)
C12—N4—Pb^ii^	123.8 (3)	C11—C10—H10	120.8
C11—N4—Pb^ii^	118.3 (3)	C9—C10—H10	120.8
N1—C1—C2	122.0 (5)	N4—C11—C10	123.0 (5)
N1—C1—C6	116.5 (4)	N4—C11—H11	118.5
C2—C1—C6	121.6 (5)	C10—C11—H11	118.5
C3—C2—C1	118.8 (5)	N4—C12—C13	122.8 (5)
C3—C2—H2	120.6	N4—C12—H12	118.6
C1—C2—H2	120.6	C13—C12—H12	118.6
C2—C3—C4	120.2 (5)	C9—C13—C12	119.4 (5)
C2—C3—H3	119.9	C9—C13—H13	120.3
C4—C3—H3	119.9	C12—C13—H13	120.3
C3—C4—C5	118.2 (5)	O3—Cl1—O2	108.6 (5)
C3—C4—H4	120.9	O3—Cl1—O4	106.9 (4)
C5—C4—H4	120.9	O2—Cl1—O4	112.7 (4)
N1—C5—C4	123.1 (5)	O3—Cl1—O5	112.5 (5)
N1—C5—H5	118.5	O2—Cl1—O5	108.5 (4)
C4—C5—H5	118.5	O4—Cl1—O5	107.7 (4)
N2—C6—C1	116.8 (4)		
			
N2—Pb—O1—C8	−10.2 (3)	C3—C4—C5—N1	−0.4 (9)
N4^i^—Pb—O1—C8	−103.3 (4)	N3—N2—C6—C1	178.8 (4)
N1—Pb—O1—C8	−26.7 (4)	Pb—N2—C6—C1	−0.9 (6)
O1—Pb—N1—C1	23.1 (4)	N3—N2—C6—C7	−2.2 (7)
N2—Pb—N1—C1	6.4 (3)	Pb—N2—C6—C7	178.1 (3)
N4^i^—Pb—N1—C1	100.1 (3)	N1—C1—C6—N2	7.1 (7)
O1—Pb—N1—C5	−166.7 (4)	C2—C1—C6—N2	−172.7 (4)
N2—Pb—N1—C5	176.6 (4)	N1—C1—C6—C7	−171.9 (4)
N4^i^—Pb—N1—C5	−89.7 (4)	C2—C1—C6—C7	8.2 (7)
O1—Pb—N2—C6	−167.9 (4)	Pb—O1—C8—N3	8.1 (6)
N4^i^—Pb—N2—C6	−84.9 (4)	Pb—O1—C8—C9	−172.5 (3)
N1—Pb—N2—C6	−2.7 (3)	N2—N3—C8—O1	3.4 (7)
O1—Pb—N2—N3	12.4 (3)	N2—N3—C8—C9	−176.0 (4)
N4^i^—Pb—N2—N3	95.4 (3)	O1—C8—C9—C13	−169.0 (5)
N1—Pb—N2—N3	177.6 (3)	N3—C8—C9—C13	10.5 (7)
C6—N2—N3—C8	167.1 (4)	O1—C8—C9—C10	12.2 (7)
Pb—N2—N3—C8	−13.1 (5)	N3—C8—C9—C10	−168.3 (4)
C5—N1—C1—C2	−0.4 (7)	C13—C9—C10—C11	−0.8 (8)
Pb—N1—C1—C2	170.4 (4)	C8—C9—C10—C11	178.0 (4)
C5—N1—C1—C6	179.8 (5)	C12—N4—C11—C10	0.3 (8)
Pb—N1—C1—C6	−9.5 (5)	Pb^ii^—N4—C11—C10	−176.6 (4)
N1—C1—C2—C3	0.2 (8)	C9—C10—C11—N4	−0.2 (8)
C6—C1—C2—C3	−179.9 (5)	C11—N4—C12—C13	0.7 (9)
C1—C2—C3—C4	−0.2 (9)	Pb^ii^—N4—C12—C13	177.4 (4)
C2—C3—C4—C5	0.2 (9)	C10—C9—C13—C12	1.7 (8)
C1—N1—C5—C4	0.5 (8)	C8—C9—C13—C12	−177.1 (5)
Pb—N1—C5—C4	−169.7 (4)	N4—C12—C13—C9	−1.7 (9)

Symmetry codes: (i) *x*−1/2, −*y*+3/2, *z*−1/2; (ii) *x*+1/2, −*y*+3/2, *z*+1/2.

### Hydrogen-bond geometry (Å, °)

**Table:**

*D*—H···*A*	*D*—H	H···*A*	*D*···*A*	*D*—H···*A*
C3—H3···O3^iii^	0.93	2.53	3.422 (10)	160
C4—H4···O1^iv^	0.93	2.45	3.277 (7)	148
C10—H10···O2^v^	0.93	2.57	3.485 (8)	170

Symmetry codes: (iii) *x*−1/2, −*y*+1/2, *z*−1/2; (iv) −*x*+1/2, *y*−1/2, −*z*+3/2; (v) −*x*+1, −*y*+1, −*z*+2.
